# The Small-Pox Status

**Published:** 1888-09

**Authors:** 


### *THE SMALL-POX STATUS.*

In our last issue we gave the fullest information obtainable in regard to this much-dreaded disease up to going to press, in order that our citizens might understand just what the health authorities were doing to prevent its spread, and how far they were succeeding in their good work.

Since that time, there have been nine new cases reported, one of which was on Fifteenth street; all the others were on the east side. One of the patients, a little girl eight years o age, died on the fourth day of the eruption at her home on Eagle street, near Cedar. Another patient who died at home, 305 Detroit street, was a child about two years old. The health authorities were not informed of this case until nine hours after death, when the Health Physician, finding the funeral in progress at the Polish church, demanded the body from the undertaker for examination. This disclosed the fact that the child had died of hemorrhagic variola, the attending physician having reported the cause of death as *purpura hemorrhagica.*

Of the seven cases taken to the Quarantine Hospital, five were of the confluent variety; one of them, a boy three years and a-half old, having died. Among them were a man and a woman who are rapidly recovering, though both had the disease in a very severe form.

At the present writing, eight patients have been discharged from the hospital cured and free from contagion, having remained under treatment an average of thirty-two days.

At the suggestion of the Health Physician, the Niagara water has been carried into the hospital, and all its appointments are convenient, comfortable, and in good sanitary condition, Dr. Le Van still continuing in charge.

To recapitulate:

| | | |
|---|---|---|
| Total number of cases of small-pox in the city to date . . | | 23 |
| Deaths . . . . . . . . . . . . . . . | 5 | |
| Discharged cured . . . . . . . . . | 8 | 13 |
| | —— | —— |
| Remaining under treatment . . . . . . . . . . . . . . | | 10 |

Of these twenty-three cases there have been—

| | | |
|---|---|---|
| Received into Quarantine Hospital . . . . . . . . . . | | 19 |
| Died . . . . . . . . . . . . . . . . | 2 | |
| Discharged cured . . . . . . . . . | 8 | 10 |
| | —— | —— |
| Remaining in hospital . . . . . . . . . . . . . . . . | | 9 |

Of these, six will be discharged in a few days.

Of the four cases not taken to hospital, three have died.

From this it will be observed that the disease is well under control of the Health authorities, and not likely to assume an epidemic form. It is well, however, to urge the timely vaccination of all who have not been thus protected, as well as the revaccination of any who are in a position to be exposed in any way to the influences of the contagion; but there need be no alarm as to the probability of its spreading to any extent. A few sporadic cases springing up here and there are to be expected in the very nature of things.

The Board of Health is to be commended upon its prompt and efficient action, whereby the disease has been so completely put in check.

